# P-1995. Diagnostic Yield of a Multiplex PCR Sample-to-Answer Array among Patients Admitted with Acute Febrile Illness, Uganda, 2019-2023

**DOI:** 10.1093/ofid/ofaf695.2160

**Published:** 2026-01-11

**Authors:** Jillian L Peters, Emmanuel Mande, Kenneth Kobba, Morgan Otita, Edgar Ndawula, Matthew Robinson, Paul W Blair, Francis Kakooza, Yukari C Manabe

**Affiliations:** Johns Hopkins University School of Medicine, Baltimore, MD; Infectious Diseases Institute, Makerere University, Kampala, Kampala, Uganda; Infectious Diseases Institute, Kampala, Kampala, Uganda; Infectious Diseases Institute, Makerere University, Kampala, Kampala, Uganda; Infectious Diseases Institute, Makerere University, Kampala, Kampala, Uganda; Johns Hopkins University School of Medicine, Baltimore, MD; Division of Infectious Diseases, Vanderbilt University Medical Center, Nashville, Tennessee; Infectious Diseases Institute, Kampala, Kampala, Uganda; Johns Hopkins University School of Medicine, Baltimore, MD

## Abstract

**Background:**

In settings with high pathogen diversity, identification of the etiology of acute febrile illness (AFI) is limited by available diagnostics. We sought to assess whether the addition of a multiplex sample-to-answer PCR-based array (mPCR) to standard microbiologic testing would increase pathogen identification among hospitalized febrile patients in Uganda.

**Figure:**
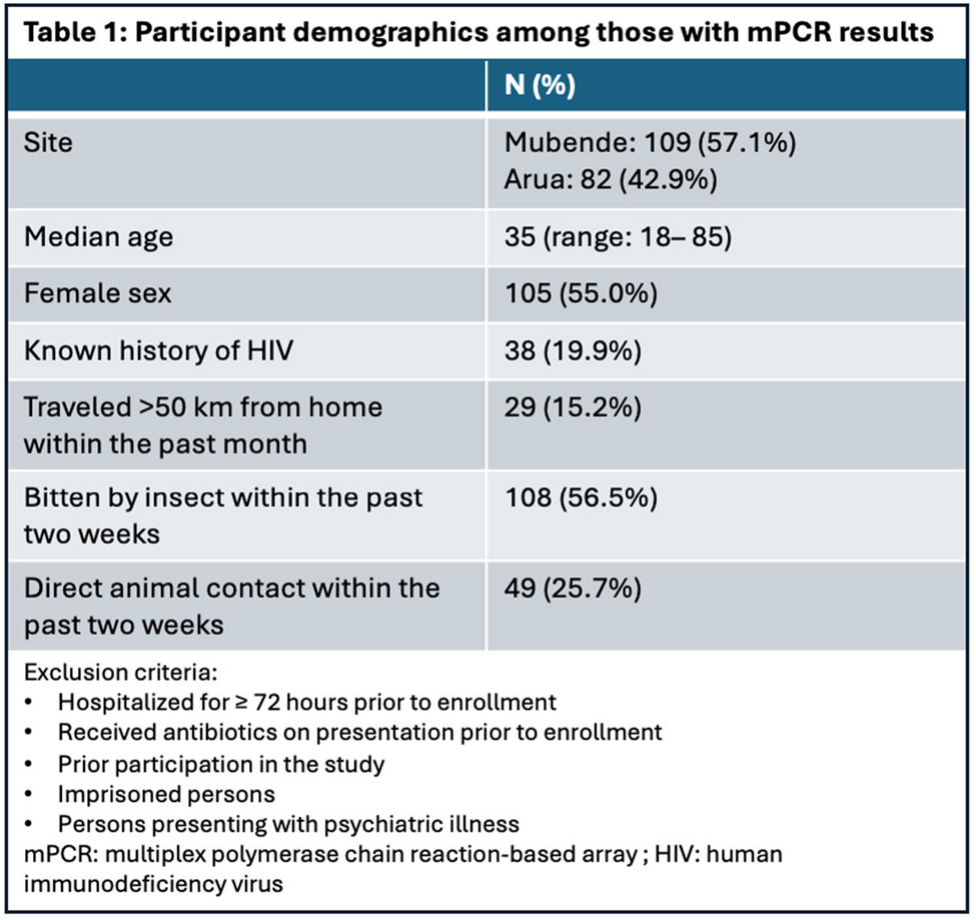

**Figure:**
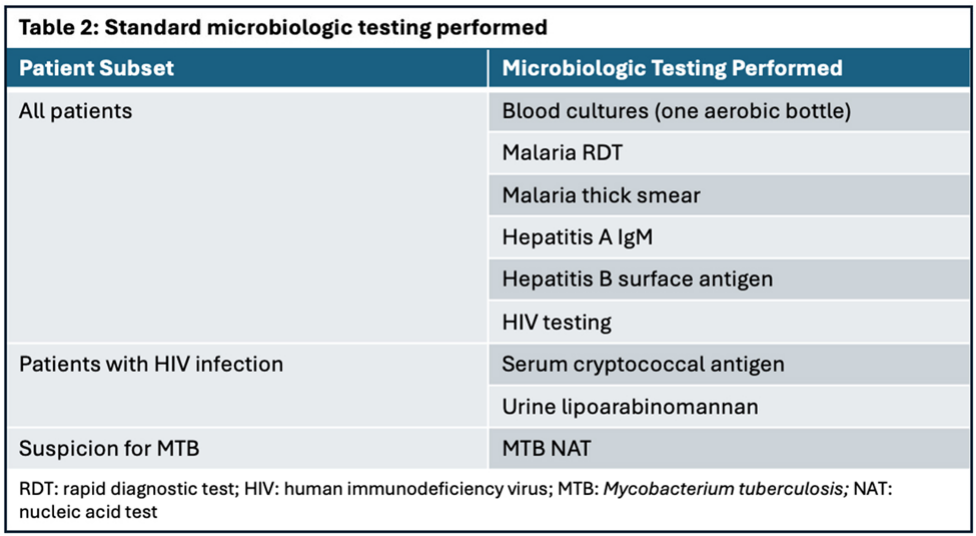

**Methods:**

Patients ≥18 years of age with fever (≥38.0°C) ≤48 hours after presentation to Mubende Regional Referral Hospital (RRH) and Arua RRH were eligible for enrollment (Table 1). Demographic, clinical, laboratory, and outcome data were collected. Protocolized standard microbiologic testing with blood cultures and rapid diagnostic tests (RDTs) was performed (Table 2). Whole blood was tested with the FilmArray Global Fever Panel – RUO (BioFire Defense, Salt Lake City, Utah), an mPCR sample-to-answer array for 9 viral, 6 bacterial, and *Plasmodium* spp.

**Figure:**
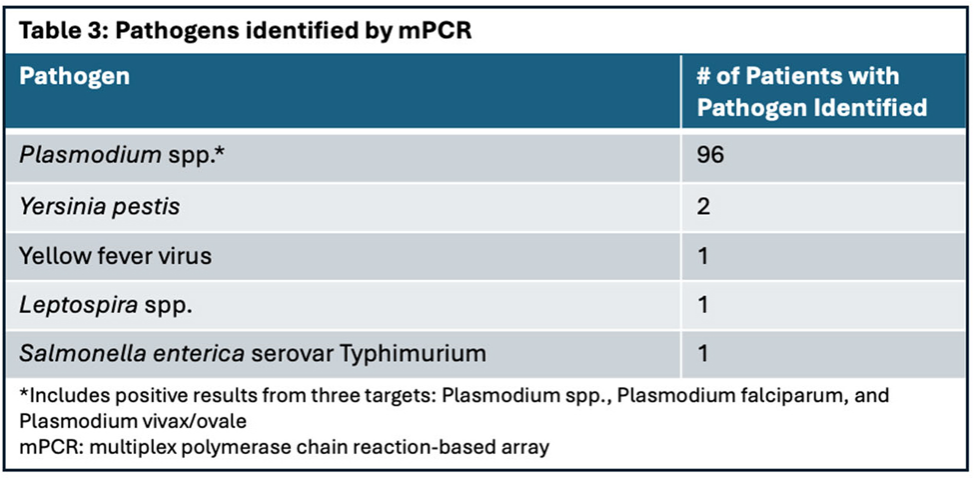

**Results:**

From August 2019 to August 2023, 283 patients were enrolled; mPCR results were available for 191 (Table 1). 58.6% (n=112 of 191) had a positive microbiologic result on standard testing. Malaria was identified in 29.8% (n=57), *M. tuberculosis* in 13.1% (n=25), hepatitis A in 7.9% (n=15), hepatitis B in 8.9% (n=17), and positive blood culture in 4.8% (n=9; 2 of 9 were possible contaminants). 10.0% (n=19) had newly positive HIV; 29.8% (n=57 of 191) had HIV at discharge; 14.0% of these (n=8 of 57) had cryptococcal antigenemia.

A positive mPCR result was identified in 44.3% (n=35 of 79) of those without a positive microbiologic result on standard testing. Two patients were positive for *Yersinia pestis*; one had malaria co-infection. One participant each was positive for yellow fever, *Leptospira* spp, and *Salmonella* Typhimurium (Table 3). Of 96 malaria-positive on mPCR (50.2%), only 49.0% (n=47 of 96) were identified on smear and/or RDT; 17% (n=10 of 57) of malaria-positive patients by smear or RDT were mPCR negative (κ=0.38, SE 0.06).

**Conclusion:**

Pathogen surveillance in AFI with mPCR increases identification of fever etiology and identifies high-consequence pathogens, enabling treatment of affected patients and intervention by public health systems. Further study of *Plasmodium* mPCR-positive febrile patients is warranted to understand clinical significance of these detections.

**Disclosures:**

Yukari C. Manabe, MD, FIDSA, FRCP, bioMerieux: Research materials to JHU|Cepheid: Research materials to JHU|Chembio: Grant/Research Support|Chembio: Research materials to JHU|Hologic: Grant/Research Support|Hologic: Research materials to JHU|Roche: Research materials to JHU

